# Evaluation of the efficacy of various remineralization agents and grape seed extract on microhardness and lesion depth of primary tooth enamel: An in vitro study

**DOI:** 10.34172/joddd.41348

**Published:** 2024-09-07

**Authors:** Pınar Serdar Eymirli, İrem Mergen Gültekin, Cansu Özşin Özler, Emel Uzunoğlu Özyürek

**Affiliations:** ^1^Department of Pediatric Dentistry, Faculty of Dentistry, Hacettepe University, Altındağ/Ankara, Turkey; ^2^Department of Pediatric Dentistry, Faculty of Dentistry, Lokman Hekim University, Altındağ/Ankara, Turkey; ^3^Department of Endodontics, Faculity of Dentistry, Hacettepe University, Altındağ/Ankara, Turkey

**Keywords:** Confocal laser scanning microscope, Grape seed extract, Microhardness, Primary tooth enamel, Remineralization

## Abstract

**Background.:**

This study evaluated the efficacy of grape seed extract (GSE) on the remineralization of primary tooth enamel alone or in combination with remineralizing agents.

**Methods.:**

The initial microhardness value of 90 primary tooth enamel samples was calculated; then, the samples were demineralized. The post-demineralization hardness of the samples was measured and the samples were randomly divided into 6 groups as follows: G1: negative control, G2: GSE, G3: NaF, G4:Casein phosphopeptide-amorphous calcium phosphate (CPP-ACP), G5: GSE+NaF, and G6: GSE+CPP-ACP (n=15). Oral environment pH cycle was applied and hardness measurements were repeated after treatments. The samples were stained with 1% rhodamine B dye and sectioned, and the lesion depth was measured. Statistical significance was set at *P*<0.05.

**Results.:**

The hardness decrease of the GSE and GSE+NaF groups was less than the other groups (*P*<0.05). The decrease was also less in the other groups than in the control group (*P*>0.05). GSE showed a positive effect when combined with NaF in maintaining microhardness but did not show the same effect when combined with CPP-ACP (*P*<0.05). Concerning penetration depth, all the groups had statistically lower values than the control group (*P*<0.05). The lowest penetration rates were observed in the GSE+NaF and GSE+CPP-ACP groups (*P*<0.05).

**Conclusion.:**

The lowest hardness decrease was observed in the GSE and GSE+NaF groups, and the lowest penetration rates were observed in the GSE+NaF and GSE+CPP-ACP groups. It has been determined that a 15% GSE solution might be used as an alternative to fluoride in primary tooth remineralization and can increase the effectiveness of fluoride when used together.

## Introduction

 The demineralization and remineralization cycles are in a balance in the oral environment. When this balance shifts in favor of demineralization, enamel destruction begins.^1^ This initial destruction of enamel is highly preventable and even reversible by providing remineralization, but dental caries remains the most common chronic disease in children and adults.^2,3^

 Poor dietary habits, xerostomia, and lack of fluoride and routine dental care are risk factors for dental caries. The importance of preventive medicine is understood day by day, and more conservative options are evaluated when intervention is required. To prevent caries formation, the patient can be given fluoride supplements, advised to reduce sugar consumption, provided with fissure sealants, and instructed in regular dental care.^4^

 Fluoride supplements have been used safely in dentistry for years. In preventing caries, the search for an alternative to fluoride continues since no agent is completely efficient, and fluoride has some dose-dependent side effects.^5^ For this purpose, agents containing casein phosphopeptide and lasers have shown efficacy as alternatives to fluoride.^6-9^

 Natural products have been used in medicine for thousands of years, and in recent years, some natural products have been introduced to prevent dental caries. One of them is grape seed extract (GSE), which contains high levels of proanthocyanidins (PA).^10,11^ It has been reported that PA strengthens collagen-based tissues by increasing collagen cross-links and accelerating the conversion of soluble collagen to insoluble collagen.^11^

 Soligo et al^12^ reported that GSE supports the remineralization of root caries^10^ and shows antimicrobial activity in root canal irrigation. There are few studies on the effect of GSE on the remineralization of tooth enamel.^13-16^ The majority of these studies were performed on permanent teeth, with very few studies on primary teeth.^15^ This study aimed to determine which agent or combination of agents is more efficient in supporting the remineralization of artificial caries in human primary teeth.

## Methods

###  Study design

 The study’s null hypothesis was that GSE shows similar remineralization support with all other remineralizing agents, and its effectiveness will not change when combined with other remineralization agents.

###  Sample preparation

 Forty-five caries- and crack-free human primary molars were selected according to sample size calculation (*P* = 0.05, statistical power = 80%). The collected teeth were extracted for exfoliation or orthodontic reasons and were visually inspected by drying with an air syringe. Teeth with visible structural defects, cavitated lesions, visible white spot lesions and/or discoloration were excluded from the study. The samples were stored in 0.10% thymol solution (ADR Group, Istanbul, Turkey) until use. The teeth were separated 1 mm below the cementoenamel junction, and root parts were removed. Two samples were obtained from 1 tooth, buccal and lingual, by separating them in the mesiodistal direction (90 enamel samples). The enamel surface of the samples was polished under water cooling with 800-, 1200-, and 2000-grit silicon carbide papers attached to the polishing device (Presi Mecapol P230, 38320 Brié et Angonnes, Grenoble, France). 3 × 3-mm windows were created on the flat surfaces of the samples, and the remaining parts were covered with two layers of nail polish. The samples were embedded in acrylic blocks with the enamel surfaces to be measured exposed. After removing the smear layer with an ultrasonic device, the average microhardness values of all the samples were measured in a microhardness test device (Shimadzu HMV-2, Tokyo, Japan).

###  Microhardness test

**Figure 1:**
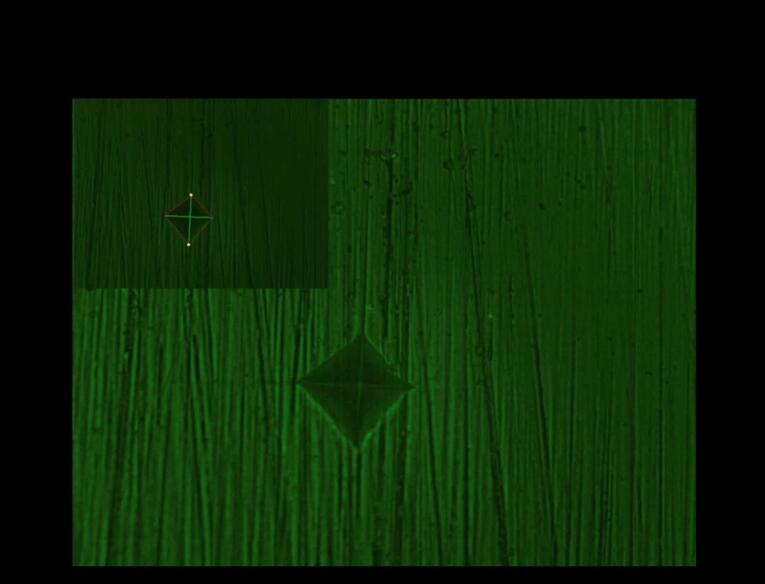
Representative penetration indentation on the enamel surface during microhardness measurement

 The microhardness values of all samples were measured using the Vickers microhardness tester by an experienced researcher (PSE) blinded to the relevant test group at three different times: at the beginning, after the artificial caries formation protocol, and at the final. The 1 × 3-mm part of the 3 × 3-mm window was marked on the acrylic block with a flame-tip bur. The samples were placed on the device table parallel to the ground, and the marked 1 × 3-mm enamel surface of the sample was analyzed with the help of the ocular piece ( × 40 magnification) on the device. Each enamel sample was impressed three times with an indentation load of 200 gf (1961 Newtons) for 10 seconds using the pyramid-shaped Vickers diamond tip of the Shimadzu HMV-2 instrument (Shimadzu HMV, Tokyo, Japan) (Figure 1). There was a distance of 100 ± 20 µm between each measurement. The average microhardness of each sample was determined by averaging these three measurements.

###  Artificial caries formation protocol

 All the samples were kept in a demineralizing solution (2.2-mM CaCl_2_.2H_2_O, 2.2-mM KH_2_PO_2_, 45-mM acetate, pH = 4.6) for 96 hours at 37 °C, as previously stated, to create artificial caries.^15^ Afterward, all the samples were washed with deionized water, and the second microhardness value was measured.

###  Preparation of grape seed extract

 Although neutral NaF gel and Casein phosphopeptide-amorphous calcium phosphate (CPP-ACP) cream are available as ready-made preparations, GSE solution is not available. There are two forms of GSE on the market. One is in capsule form (Solgar, Leonia, USA), and the other is in liquid form (Immu-nat, Muğla, Turkey). The total phenolic content (TPC) of both forms of GSE was measured. The measurements were compared to a standard curve of prepared gallic acid solution (5, 10, 20, 40, 60, 80, and 100 ppm) and expressed as grams of gallic acid equivalents (GAE) per mL of the extract (total dry matter = 31.72%). It was found to be 60 mg of GAE/mL for the liquid extract or 190 mg of GAE/g in terms of dry matter. The same data were found as 45 mg of GAE/mL or 142 mg of GAE/g for the capsule with the same dry matter. Liquid extract was preferred because the TPC is higher. The type of grape used in the liquid extract is ‘vitis vinifera’. 15 mL of the extract was completed to 100 mL with distilled water, and 15% GSE solution was obtained.

###  Treatments and pH Cycling

 The samples were randomly assigned to 6 groups (n = 15). The remineralization procedure determined for each group was applied (Table 1). After applying the remineralizing agents, the samples were wiped with a sterile sponge and kept in artificial saliva for two hours. Then, the pH cycling was applied. The samples were kept in the demineralizing solution for 30 minutes and then washed with deionized water. It was kept in a neutral-pH solution (20-mM HEPES; 2.25-mM CaCl_2_·2H_2_O; 1.35-mM KH_2_PO_4_; 130-mM KCl; pH = 7.0) for 10 minutes and then washed with deionized water. This cycle was repeated six times daily for eight days to simulate the oral environment. The remineralization procedures were repeated once a day for eight days, during which the cycle was applied and then the cycle was started. All the samples were kept in solution at neutral pH overnight. The samples of each group were kept in separate containers, and the solutions were prepared freshly every two days.

**Table 1:** Experimental groups and treatments

	**Treatment**	**Method of Application**
Control	Negative Control	Remineralization procedure not applied
GSE	15% GSE solution	10 minutes in solution
NaF	2% (9000 ppm) neutral NaF gel	4 minutes with an applicator
CPP-ACP	10% CPP-ACP (CPP-ACP, GC Tooth Mousse, GC Corp., Tokyo, Japan)	3 minutes with an applicator
GSE + NaF	2% neutral NaF gel, then 15% GSE solution	4 minutes with an applicator, then 10 minutes in solution
GSE + CPP-ACP	10% CPP-ACP, then 15% GSE solution	3 minutes with an applicator, then 10 minutes in solution

GSE, grape seed extract; CPP-ACP: Casein phosphopeptide-amorphous calcium phosphate

 After the 8-day pH cycle, the samples were washed with deionized water for 2 minutes, and then the microhardness measurements were repeated.

###  Confocal laser scanning microscopy (CLSM)

**Figure 2:**
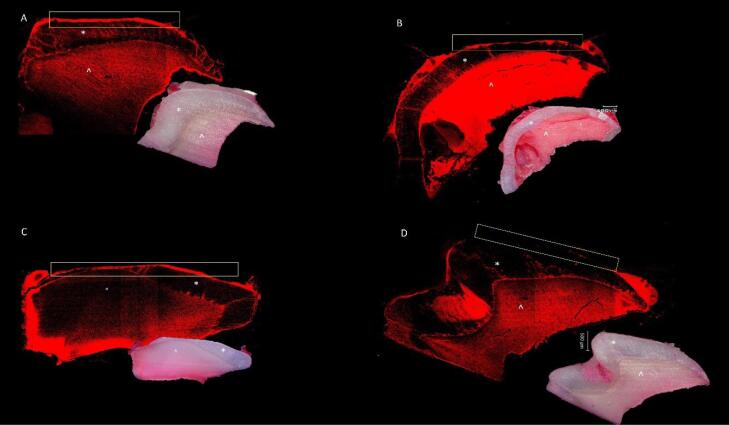
Stereomicroscope and confocal laser microscope images of representative samples. (A) Negative control; the deepest penetration values were measured in the control group. (B) NaF. (C) CPP-ACP; different penetration values were measured. (D) GSE + NaF; the representative sample has almost no penetration. The yellow rectangle indicates the area of penetration in enamel. * denotes the enamel, and ^ denotes the dentin of the samples [scale bar of stereomicroscopic images is 400 µm (B) and scale bar of CLSM images is 500 µm (D)]

 The remaining 3 × 2-mm part of each enamel fragment was sectioned longitudinally across the lesions with a low-speed water-cooled diamond saw (Isomet 1000, Buehler, Lake Bluff, IL, USA) to obtain 1-cm-thick sections. The samples were stained with 0.1-mM rhodamine B solution (Aldrich Chemical Co., Milwaukee, WI, USA) overnight, dried with a sterile sponge, and observed with CLSM (LSM 510; Carl Zeiss, Jena, Germany). Digital images were taken, and the penetration depth of rhodamine B solution into the remineralized enamel was measured with the CLSM image browser (Carl Zeiss, Germany) (Figure 2). Penetration values were measured by a researcher blinded to the groups (CÖÖ). For measurement consistency, the same researcher measured the values twice with an interval of 10 days.

###  Statistical analysis

 The data were analyzed using the SPSS program (Version 23, IBM, Armonk, NY, USA). Microhardness changes were compared with repeated-measures two-way ANOVA and post hoc Bonferroni test. A dependent t-test was used to compare the averages of penetration depth values of rhodamine B to evaluate the measurement consistency (*P* < 0.05). The penetration depth of rhodamine B solution was compared with one-way ANOVA and post hoc Bonferroni test (*P* < 0.05).

## Results

###  Microhardness change 

 After measuring the initial microhardness values, the samples were demineralized, and their microhardness values were re-measured. Table 2 presents the percentage decreases in microhardness after demineralization. A mean reduction of 71.77‒78.07% was observed in all groups, with no significant difference between the groups (*P* > 0.05), indicating that the demineralization process was successful in all groups, and the percentage of microhardness decrease in each group compared to the initial was homogeneous. When evaluating the microhardness reduction, this formula was used:


$$Microhardness\;reduction\left(\%\right)=\frac{\left(Demineralizarion\;or\;Remineralization\;microhardenss\;measurement-Initial\;microhardness\;measurement\right)}{Initial\;Microhardness\;measurement}\times 100$$

**Table 2:** Microhardness reduction percentage (%) values compared to initial measurements at two intervals as mean and standard deviation

**Groups**	**Difference between the measurements (%) before and after demineralization **	**Difference between measurements (%) before and after remineralization +pH cycling **
Control	77.17 ± 5.12a*	91.71 ± 3.11b*
GSE	75.83 ± 8.19a*	82.67 ± 3.67b^ +
NaF	75.43 ± 11.10a*	88.55 ± 5.15b*^
CPP-ACP	71.77 ± 15.28a*	86.79 ± 8.71b*^
GSE + NaF	74.56 ± 9.75a*	80.11 ± 5.64b +
GSE + CPP-ACP	78.07 ± 6.37a*	88.16 ± 6.07b*^

GSE, grape seed extract; CPP-ACP: Casein phosphopeptide-amorphous calcium phosphate. a-b shows the differences between columns in the same row (differences between microhardness values in the same group). *^ + show differences between rows in the same column (differences among groups in the same measurement period).

**Figure 3:**
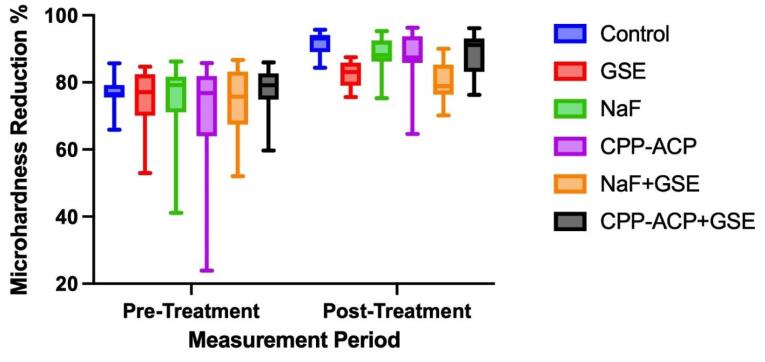
Box plots show the percentage decreases in Vickers microhardness values of the samples at different measurement intervals

 Then, remineralization procedures were applied to the experimental groups, and all the samples were exposed to a pH cycling procedure for eight days. After this period, the microhardness values were re-measured, and the reduction relative to the initial values was calculated again. Table 2 and Figure 3 present the changes in microhardness values after the remineralization processes. Since the pH cycling procedure was applied to all the groups after the remineralization process, microhardness values continued to decrease in all the groups. It has been observed that this decrease varies between 80.11% and 91.71% on average. The amount of reduction was found to be statistically significant for all the groups (*P* < 0.05).

 When the effectiveness of the applied remineralization solutions was evaluated, it was observed that the most effective results were obtained with GSE + NAF and GSE solutions, and these two groups were statistically different from the control group (*P* < 0.05). GSE showed a positive effect when combined with NaF in maintaining microhardness but did not show the same effect when combined with CPP-ACP (*P* < 0.05). Although the lower reduction results were obtained with GSE when used alone, no significant difference was found between GSE, NaF, and CPP-ACP (*P* > 0.05) (Figure 3).

###  Penetration depth

 There was no difference between the means of the groups at two different measurement times (*P* > 0.05). The final values were then determined using the average of two different measurements. Table 3 presents the penetration values. Penetration values varied between 30.99 and 200.71 µm on average in the groups. The lowest penetration rates were observed in the GSE + NAF and GSE + CPP-ACP groups, with a significant difference from the other groups (*P* < 0.05). Rhodamine B penetration values measured as a result of the application of the solutions alone were statistically indistinguishable from each other (*P* > 0.05), which were significantly lower than the control group (*P* < 0.05). The highest penetration values were measured in the control group, and these values were significantly higher than all the other groups (*P* < 0.05) (Figure 2).

**Table 3:** Penetration depth of rhodamine B in micrometers (mean and standard deviation)

**Groups**	**Depth of penetration**
Control	200.71 ± 45.95^a^
GSE	122.26 ± 33.57^b^
NaF	107.14 ± 30.26^b^
CPP-ACP	144.32 ± 43.82^b^
GSE + NaF	30.99 ± 22.52^c^
GSE + CPP-ACP	49.75 ± 23.28^c^

GSE, grape seed extract; CPP-ACP: Casein phosphopeptide-amorphous calcium phosphate Different superscripts show statistical differences (*P* < 0.05).

## Discussion

 Topical fluorides and CPP-ACP are common agents used to prevent dental caries. The mechanism of action of fluoride application involves replacing the rapidly dissolving hydroxyl ion with the fluorine ion in the face of acid attacks, leading to fluorapatite formation and more resistance against acid attacks.^17^ It has been reported that CPP-ACP effectively prevents caries by stabilizing calcium and phosphate on the tooth surface.^7^ Fluoride agents in different contents and forms and CPP-ACP have proved effective in preventive dentistry.^7,8,18,19^ The effectiveness of GSE in remineralization is a current issue that has attracted attention. It has been suggested that it can stabilize the collagen matrix better than other agents by establishing covalent, hydrogen, and ionic bonds with collagen due to multiple hydroxyl groups in its structure.^20^ However, most studies have focused on dentin remineralization.^10,21-25^ While the number of studies examining the effect of GSE on enamel remineralization is quite limited, only one study evaluated primary tooth enamel.^15^ The effectiveness of GSE on the remineralization of primary tooth enamel was compared with NaF and CPP-ACP in the current study, and its effectiveness when combined with these agents was also evaluated.

 Polyphenols and proanthocyanidins, which are primarily condensed tannins, are the biologically active components of GSE.^26^ The Folin-Ciocalteu method^27^ was used to determine the TPC of the GSE used in this study, which was 190 mg GAE/g. It has been reported that the TPC of GSE used in a similar study was higher than the present study.^15^ GSE must be extracted from grape seed to make a one-to-one comparison with other studies. However, in most studies, ready-made preparations were used, as in the current study, and an estimated calculation was made based on the amount of dry matter.^10,16,22,23^ Furthermore, the TPC value of the extract may vary depending on the type of grape to be extracted, the extraction method, and the solvents used.

 An examination of studies in the literature showed that the GSE solution was used at various concentrations. The most common concentration was 6.5%.^10,21,22^ The effectiveness of different concentrations of GSE solution was evaluated in a study, and the best results were found with 15% concentration.^23^ GSE was also used at a concentration of 15% in the current study. Yassen and Safy^28^ reported that the 15% GSE solution increased the mechanical properties of dentin, similar to the 1000-ppm NaF solution. It has also been reported that GSE could be a new and promising alternative for treating carious lesions.^29^

 In addition to different concentrations of GSE, different application durations were used in the literature. Different application times were used for the GSE, such as 20, 30, 60, and 120 seconds, 10 and 30 minutes, and 1, 2, and 4 hours.^30-35^ It has been reported that the hardness value of demineralized dentin increases as the exposure time to PA increases.^30^ Liu et al.^35^ investigated the resistance of dentin collagen to enzymatic degradation by associating it with the percent weight loss. They suggested that at even as little as 10 seconds, GSE application could increase the resistance of collagen to enzymatic degradation, which might have occurred through a chemical mechanism between collagen and PA. Bedran-Russo et al^30^ applied the GSE solution for 10 minutes, 30 minutes, 1 hour, 2 hours, and 4 hours and reported that the highest average hardness values were obtained after 10 minutes. Considering this information, a 10-minute application was preferred in the present study.

 In this study, microhardness and confocal laser scanning microscope (CLSM) examinations were used to evaluate the remineralization of primary tooth enamel. When remineralizing agents were used alone, microhardness measurements revealed that the samples remineralized with GSE had the highest hardness values. In a study by Xie et al,^10^ 1000-ppm NaF and 6.5% GSE solution showed similar results in terms of microhardness values. The better results of the GSE solution in the present study could be due to the differences in concentrations and the samples (permanent teeth root dentin vs. primary tooth enamel). In a study in which 12.5% GSE solution was applied to primary teeth, a statistically significant difference was found between the control group regarding microhardness values.^15^ In the same study, amorphous clusters, which are GSE reaction products, were seen in the SEM images of the GSE-exposed samples. These clusters were thought to be scaffold deposits that aid in enamel remineralization.^15^ Studies have shown that GSE positively affects the demineralization and/or remineralization process by increasing collagen cross-links on dentin caries.^10,30,32^ GSE induces collagen cross-links by interacting with proteins through four different mechanisms: covalent, ionic, hydrogen bonding, and hydrophobic interaction.^10,32^ PA-based compounds have a high affinity for proline-rich proteins such as collagen. Proline-PA complexes form rapidly in collagen-rich tissues such as dentin.^32^ Acil et al^36^ reported a small amount of type 1 collagen in enamels. In addition, studies have determined that type X collagen is thought to play a role in enamel mineralization.^37^

 The increased fluorescence on CSLM examination indicates demineralization of formed enamel carious lesions. The thickness of the fluorescent band (depth of penetration) is directly related to the porosity of the demineralized enamel and the amount of dye penetrating the pores. The groups with the lowest penetration values were the NaF and CPP-ACP groups combined with GSE. The highest penetration was seen in the control group. When used alone, there was no significant difference between the NaF, GSE, and CPP-ACP groups. In the study of Zhao et al.,^16^ different concentrations of GSE solution and 10-ppm NaF were applied to bovine enamel, and according to the CSLM results, the NaF group exhibited the best results in enamel remineralization. In contrast, the 2-mg/mL and 3-mg/mL GSE groups yielded better results compared to the control group. In this study, the combination of GSE and NaF was not applied, and the NaF group showed the best remineralization compared to GSE. The present study found no significant difference between these two groups. It was thought that this difference between the two studies might be due to the solution concentrations and the used enamel samples.

## Conclusion

 Based on all these results, no agent or combination of agents could be found in the remineralization of deciduous tooth enamel that would completely resist the acid attacks that occur during the day while also not decreasing the enamel microhardness. However, according to the results of this study, the remineralization agent that provided the most negligible decrease in the microhardness of primary tooth enamel was NaF combined with GSE. While the mechanisms of action of GSE and NaF on enamel are completely different, they produced the best results due to a synergistic effect when used together. It was concluded that GSE was also effective in microhardness when applied alone. The NaF and CPP-ACP groups combined with GSE produced the lowest values regarding lesion depth. Based on all these data, GSE could be an alternative natural agent to fluorides, and maximum effectiveness can be achieved by combining it with NaF.

## Acknowledgments

 The authors would like to thank Sevilay Karahan for the statisticalanalysis. The current study was supported bytheHacettepe University Scientific Research Project CoordinationUnit (Project Number: TSA-2018-17520).

## Competing Interests

 No potential conflict of interest relevant to this article was reported.

## Ethical Approval

 Ethical approval of the study was granted by Hacettepe University’s Non-interventional Ethics Committee (Decision no: GO 20/779).
